# Effect of Fish Oil vs. Resolvin D1, E1, Methyl Esters of Resolvins D1 or D2 on Diabetic Peripheral Neuropathy

**DOI:** 10.4172/2155-9562.1000453

**Published:** 2017-12-24

**Authors:** Alexander Obrosov, Lawrence J Coppey, Hanna Shevalye, Mark A Yorek

**Affiliations:** 1 Department of Internal Medicine, University of Iowa, Iowa City, USA; 2 Department of Veterans Affairs, Iowa City Health Care System, Iowa City, USA; 3 Veterans Affairs Center for the Prevention and Treatment of Visual Loss, Iowa City, USA; 4 Fraternal Order of Eagles Diabetes Research Center, University of Iowa, Iowa City, USA

**Keywords:** Omega-3 polyunsaturated fatty acids, Fatty acid metabolism, Diabetic neuropathy, Resolving, Epidermal nerve fibers, Corneal nerve fibers, Type 2 diabetes

## Abstract

**Objective:**

Fish oil is enriched in omega-3 polyunsaturated fatty acids primarily eicosapentaenoic and docosahexaenoic fatty acids. Metabolites of these two polyunsaturated fatty acids include the E and D series resolvins. Omega-3 polyunsaturated fatty acids and resolvins have been reported to have anti-inflammatory and neuroprotective properties. The objective of this study was to evaluate the efficacy of menhaden oil, a fish oil derived from the menhaden, resolvins D1 and E1 and the methyl esters of resolvins D1 and D2 on diabetic peripheral neuropathy. Hypothesis being examined was that the methyl esters of resolvins D1 and D2 would be move efficacious than resolvins D1 or E1 due to an extended half-life.

**Methods:**

A model of type 2 diabetes in C57BL/6J mice was created through a combination of a high fat diet followed 8 weeks later with treatment of low dosage of streptozotocin. After 8 weeks of untreated hyperglycemia type 2 diabetic mice were treated for 8 weeks with menhaden oil in the diet or daily injections of 1 ng/g body weight resolvins D1, E1 or methyl esters of resolvins D1 or D2. Afterwards, multiple neurological endpoints were examined.

**Results:**

Menhaden oil or resolvins did not improve hyperglycemia. Untreated diabetic mice were thermal hypoalgesic, had mechanical allodynia, reduced motor and sensory nerve conduction velocities and decreased innervation of the cornea and skin. These endpoints were significantly improved with menhaden oil or resolvin treatment. However, the methyl esters of resolvins D1 or D2, contrary to our hypothesis, were generally less potent than menhaden oil or resolvins D1 or E1.

**Conclusion:**

These studies further support omega-3 polyunsaturated fatty acids derived from fish oil via in part due to their metabolites could be an effective treatment for diabetic neuropathy.

## Introduction

Peripheral neuropathy affects about 50% of the diabetic population and no effective treatment is available. Our laboratory has shown that treating type 1 or type 2 diabetic rodents with menhaden oil can delay progression and with late intervention reverse many endpoints related to peripheral neuropathy [1–5]. Moreover, we have demonstrated in studies using type 1 and type 2 diabetic mice that daily treatment with resolvin (resolution phase interaction products) D1 improves peripheral neuropathy [6,7].

Resolvins and neuroprotectin D1, metabolites of eicosapentaenoic and docosahexaenoic acids found in fish oil, have anti-oxidant, anti-inflammatory and neuroprotective properties [8,9]. E-series resolvins are oxygenated metabolites of eicosapentaenoic acid and D series resolvins are derived from docosahexaenoic acid. Neuroprotectin D1 is synthesized from docosahexaenoic acid requiring the enzyme 15-lipoxygenase-1. Neuroprotectin D1 produced following treatment with docosahexaenoic acid of corneas damaged by refractive surgery has been shown to have nerve regenerating properties [10,11]. Treating primary cultures of trigeminal ganglia neurons from Swiss Webster mice also increases neurite outgrowth [10]. We have reported that neurite outgrowth by dorsal root ganglia neuron from C57Bl6/J mice was increased by resolvin D1 [6]. However, whether resolvin E1 promotes neurite outgrowth or provides efficacy toward diabetic peripheral neuropathy is unknown. Both eicosapentaenoic and docosahexaenoic acids are present in fish oil and other marine products and determining if both provide similar protection toward diabetic peripheral neuropathy is an important issue to address. Methyl esters of resolvins have a longer biological half-life, thus it is also important to determine if these metabolites are more efficacious in vivo for diabetic peripheral neuropathy [12]. Thus, in this study we investigated whether resolvin E1 can attenuate diabetic peripheral neuropathy and if the methyl esters of resolvins D1 or D2 have a greater efficacy than resolvin D1 on diabetic peripheral neuropathy.

Identifying a modified metabolite of docosahexaenoic acid with a longer half-life that could be administered daily could be a preferred approach for some human subjects compared to the daily consumption of fish oil capsules due to the gastric side effects considered unpleasant by some individuals such as belching.

## Materials and Methods

### Materials

Unless stated otherwise all chemicals used in these studies were obtained from Sigma-Aldrich Co. (St. Louis, MO).

### Animals and diet fatty acid composition analysis

C57BL/6J mice were obtained from Jackson Laboratories. Mice were housed in a certified animal care facility and water and standard diet were provided ad libitum. Measures were taken to minimize pain or discomfort and all experiments were conducted in accordance with the Public Health Service Policy on Humane Care and Use of Laboratory Animals and were compliant with all institutional guidelines for use of animals (IACUC approval 5071451). Twelve week old C57BL/6J mice were divided into seven groups. After 1 week on a standard diet (3.0 kcal/g, 13% kcal fat, 7001, Harlan Teklad, Madison, WI) six of the groups were fed a high fat diet for eight weeks (5.2 kcal/g, 60% kcal fat, D12492; Research Diets, New Brunswick, NJ). The group maintained on the standard diet served as the control group (I) and was fed the standard diet for the duration of the study. To create a model for type 2 diabetes, these mice were treated with 100 mg/kg streptozotocin, i.p. (EMD Chemicals, San Diego, CA). Three days later, if the blood glucose was less than 13.8 mM (250 mg/dL), a second dose of streptozotocin (50 mg/kg) was administered (Accu-Chek, Roche Inc., Indianapolis, IN) [13]. Mice with a blood glucose ≥ 13.8 mM (250 mg/dL) 1 week after the initial injection of streptozotocin were considered diabetic. Following 8 weeks of hyperglycemia, one group of mice was continued on the high fat diet (non-treated diabetic group, (II)). A second group (III) was fed a high fat diet with ½ of the lard-derived calories replaced with menhaden oil (Research Diets D10122003). The four other groups remained on the high fat diet and were treated with resolvin D1 (7S, 8R, 17S-trihydroxy-4Z,9E,11E,13Z,15E,19Z-docosahexaenoic acid, (IV)), resolvin E1 (5S, 12R, 18R-trihydroxy-6Z,8E,10E,14Z,16E-eicosapentaenoic acid, (V)), 17(R)-resolvin D1 methyl ester (7S,8R, 17R-trihydroxy-4Z,9E,11E,13Z,15E,19Z-docosahexaenoic acid, methyl ester, (VI)) or resolvin D2 methyl ester (7S,16R,17S-trihydroxy-4Z,8E, 10Z,12E,14E,19Z-docosahexaenoic acid, methyl ester) VII)) (Cayman Chemical Company, Ann Arbor, MI). All resolvin compounds were dissolved in 0.4% ethanol and the mice received daily i.p. injections of 1 ng/g body weight. The selection of 1 ng/g as the dose for the resolvins used in this study was based on 1 ng/g of resolvin D1 providing maximal effects in a previous study [6]. The control and non-treated diabetic group also received daily injections of 0.4% ethanol as a vehicle control. The treatment phase lasted for 8 weeks.

Diets and liver samples were used to determine the fatty acid composition by gas-liquid chromatography. Following extraction of the lipids with a 2:1 (vol/vol) mixture of chloroform and methanol, phase separation was induced with a solution of 154 mM NaCl and 4 mM HCl. The chloroform/lipid fraction was trans esterified using 14% boron trifluoride in methanol and the fatty acid methyl esters extracted into heptane before separation by gas-liquid chromatography [14]. Individual fatty acids peaks as % of total fatty acids present were identified by comparison to known fatty acid standards. The fatty acid composition of the standard diet (Harlan Teklad 7001), high fat diet (Research Diets D12492) and the custom prepared menhaden oil supplemented high fat diet (Research Diets D10122003) is provided in Table 1. As expected the levels of eicosapentaenoic and docosahexaenoic acids are increased in the diets containing menhaden oil.

Behavioral examinations: Thermal sensitivity was measured using the Hargreaves method with instrumentation provided by IITC Life Science; Woodland Hills, CA (model 390G). This procedure was initiated by placing the mouse in the observation chamber. The mouse was allowed to acclimate to the warmed glass surface (300C) and surroundings for a period of 15 min. Afterwards, the heat source was maneuvered so that it was under the heel of the hind paw, activated, a process that turns on a timer and locally warms the glass surface and when the mouse withdrew its paw, the timer and the heat source was turned off [13]. Following an initial recording, which was discarded, four measurements were made for each hind paw, with a rest period of 5 min between each examination. The mean of the measurements, reported in seconds, was used as a measure of the thermal nociceptive response latency. Mechanical allodynia was evaluated by quantifying the withdrawal threshold of the hind paw in response to stimulation with flexible von Frey filaments as previously described [15]. The data were reported in grams. The tactile response tests were repeated at least three times with a rest period of 10 min between tests. The behavioral examinations were performed in a masked fashion on different days and completed immediately before the terminal procedures.

Motor and sensory nerve conduction velocity: Mice were anesthetized with Nembutal (75 mg/kg, i.p., Abbott Laboratories, North Chicago, IL). Motor and sensory nerve conduction velocities were assessed as in previous experiments [16]. Body temperature was monitored using a rectal probe and regulated between 36°C and 37°C using a heating pad and radiant heat. This procedure maintained a normal temperature near the sciatic nerve [2]. Motor nerve conduction velocity was calculated by using the stimulus artifact of the evoked potential, subtracting the latency measurement (in milliseconds) from the sciatic notch from the latency measurement of the Achilles tendon and dividing the difference by the distance between the two stimulating electrodes (measured in millimeters). Sensory nerve conduction velocity equaled the distance between stimulating and recording electrodes over the latency to initial peak negative deflection. Both motor and sensory nerve conduction velocity was reported in m/s.

Corneal nerve imaging: The Rostock cornea module for the Heidelberg Retina Tomograph (Heidelberg Engineering, Vista, CA) was used for in vivo assessment of sub-epithelial nerves in the mouse cornea as described previously [6,17]. Briefly, anesthetized mice were fitted to a stereotaxic mouse head holder (model 921-E; David Kopf Instruments, Tujunga, CA) and secured to a platform that allows for three-dimensional adjustments. GenTeal eye lubricant gel (Alcon; Fort Worth, TX) was applied to the lens and advanced forward to make contact with the mouse cornea epithelium. At least three non-overlapping images of the sub-epithelial nerves were acquired per mouse and assessed for total nerve length per image. Corneal nerve fiber length has proven to be the best morphological parameter in diagnosing diabetic neuropathy showing the lowest coefficient of variation [18]. Corneal nerve fiber length is represented as a mean value of the nerve lengths measured from the images and expressed in mm/mm^2^.

Intraepidermal nerve fiber density in the hind paw: Skin was collected from the footpads for determination of intraepidermal nerve fibers as in previous experiments [6,17]. Nerve profiles were imaged using a Zeiss LSM710 confocal microscope with a 40× objective (EC Plan-Neofluar 40x/0.75), counted by two independent investigators masked to the sample condition and profiles were normalized to the length of the epidermis in millimeters.

Analysis of biological markers: We used a glucometer (Accu-Chek, Roche Inc., Indianapolis, IN) to measure blood glucose in a non-fasting mouse. Steatosis was examined by freezing liver samples in OCT compound (Sakura FineTek USA, Torrance, CA). Afterwards, 5 μm thick sections were incubated with BODIPY (Molecular Probes, Carlsbad, CA, USA), at a 1:5000 dilution in 1.0% BSA for 1h at room temperature. The samples were washed, mounted using ProLong^®^ Gold antifade reagent (Molecular Probes, Carlsbad, CA, USA) and covered with a glass coverslip. Images of each liver section were collected using Zeiss 710 LSM confocal laser scanning microscope. These images were analyzed for % area fraction of lipid droplets using Image J software. Chymotrypsin-like proteasome activity was assayed in liver extracts using 96-well format as described by Otoda et al. [18,19]. The reaction mixture contained 100 μg of liver extract protein, 100 μM of peptide substrate Suc-Leu-Leu-Val-Tyr-AMC in an assay buffer consisting of 50 mM HEPES (pH 7.8), 10 mM NaCl, 1.5 mM MgCl_2_, 1 mM EDTA, 1 mM EGTA, 250 mM sucrose, 5 mM dithiothreitol, 2 mM ATP. The proteasome inhibitor MG132 was added at 20 μM concentration for a background control for each sample and purified murine 20S proteasome (Boston Biochem, Cambridge, MA. USA) served as a positive control in each plate. Free AMC fluorescence was measured using a 355/460 nm filter set in FLUOstar Optima microplate reader (BMG Labtech, Cary, NC, USA). The proteasome activity was expressed in U/mg protein with 1 U equal to 1 nmol of AMC released per 1 min. Protein concentration for each liver sample was measured with the bicinchoninic acid protein assay (Thermo Fisher Scientific, Waltham, MA). Blood was collected and serum obtained for determination of free fatty acid, triglyceride, free cholesterol and resolvin D1 using commercial kits from Roche Diagnostics, Mannheim, Germany; Sigma-Aldrich Co., St. Louis, MO; BioVision, Mountain View, CA; and Cayman Chemical Co., Ann Arbor, MI respectively. Serum thiobarbituric acid reactive substances levels were also determined as a marker of oxidative stress as previously described [20]. Briefly, 200 μL of serum was heated to boiling in 0.75 mL of phosphoric acid (0.19 M), 0.25 ml thiobarbituric acid (0.42 mM) and 0.3 mL water for 60 min. Afterwards, methanol/NaOH was used to precipitate each sample, which was then centrifuged for 5 min. Supernatant was obtained and fluorometric analysis performed at excitation wavelength of 532 nm and emission wavelength of 553 nm. Standards were prepared by the acid hydrolysis of 1,1,3,3-tetraethoxypropane. The data were reported as mg/ml serum.

Data Analysis: Results are presented as mean ± S.E.M. Comparison between control, non-treated and treated diabetic mice were conducted using one-way ANOVA and Bonferroni post-hoc test comparison (Prism software; GraphPad, San Diego, CA). A P value of less than 0.05 was considered significant.

## Results

For these studies mice were made diabetic with a streptozotocin low dose treatment strategy following 8 weeks on a high fat diet. After 8 weeks of hyperglycemia mice were treated daily with menhaden oil or exogenously with resolvin D1, E1 or methyl esters of resolvins D1 or D2 for 8 weeks and then analyzed as described below.

Effect on weight, blood glucose, serum lipid and resolvin D1 levels, serum thiobarbituric acid levels and liver proteasome activity and steatosis. Data in Table 2 demonstrate that all mice at the beginning of the study weighed approximately the same. At the beginning of treatments the diabetic mice generally weighed more than control mice although the difference was not significant for all groups. Blood glucose levels were significantly increased in all groups of diabetic mice at the beginning of treatment. During the treatment phase all mice gained weight and generally all diabetic mice weighed more than the control mice although the difference was not significant for all groups. At the end of the study the blood glucose levels of all diabetic mice were significantly higher than control mice and were similar to the blood glucose levels at the beginning of the treatment phase for all groups of mice and were not impacted by treatments.

At the end of the study, serum triglycerides levels were increased in the non-treated diabetic mice compared to control mice but the difference was not significant. Treating diabetic mice with menhaden oil in the diet or exogenously with resolvins D1 and E1 or the methyl esters of resolvins D1 and D2 reduced triglyceride levels to control values or lower. The level of serum triglyceride in diabetic mice treated with menhaden oil or the methyl esters of resolvins D1 or D2 were significantly lower than in non-treated diabetic mice. Serum free fatty acid levels were significantly increased in non-treated diabetic mice compared to control mice and was corrected with all treatments.

Serum free cholesterol levels were significantly increased in non-treated diabetic mice compared to control mice, which was not corrected by treatment with menhaden oil in the diet or resolvin D2 methyl ester and only partially corrected by exogenous treatment with resolvins D1, E1 and D1 methyl ester. Serum levels of resolvin D1 were significantly increased in diabetic mice treated with a diet enriched with menhaden oil compared to control mice and untreated diabetic mice (Table 2). Serum resolvin levels were not determined in diabetic mice treated with resolvin D1 because previous studies had shown that treatment did not cause an increase in serum levels of resolvin likely due to the short half-life of resolvins in circulation [6,7].

Serum thiobarbituric acid levels, a marker for oxidative stress, were significantly increased in untreated diabetic mice (Table 2). Treating diabetic mice with dietary menhaden oil did not improve serum thiobarbituric acid levels. However, exogenous treatment with resolvins did lower serum thiobarbituric acid levels.

For a marker of endoplasmic reticulum stress we examined liver proteasome activity [19]. Liver proteasome activity was decreased in non-treated diabetic mice compared to control mice and was partially improved by dietary enrichment with menhaden oil or treatment with methyl ester of resolvin D2 (Table 2). Treating diabetic mice with resolvins D1, E1 or D1 methyl ester had minor to no impact on improving liver proteasome activity. Liver steatosis (fatty liver) was significantly increased in non-treated diabetic mice (Table 2). Treating diabetic mice with a diet enriched with menhaden oil or with daily injections of resolvins significantly reduced liver steatosis. However, liver steatosis remained significantly increased in all treated diabetic mice compared to control mice.

Effect on liver fatty acid composition. Data in Table 3 provide the fatty acid composition of liver from control, non-treated diabetic and diabetic mice treated with menhaden oil in the diet. The fatty acid composition profile of liver from diabetic mice fed the high fat diet was not noticeably different from the fatty acid composition of liver from control mice. However, in diabetic mice treated with menhaden oil the content of arachidonic acid was significantly decreased while the content of eicosapentaenoic and docosahexaenoic acid was significantly increased. Treatment of diabetic mice with resolvin D1, E1 or methyl esters of resolvins D1 or D2 did not impact the fatty acid composition of the liver compared to untreated diabetic mice (data not shown).

**Figure 1:**
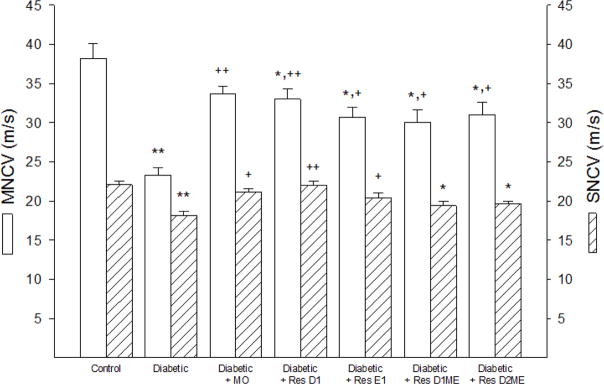
Effect of Menhaden oil dietary enrichment or daily treatment with resolvin D1, E1, D1 methyl ester or D2 methyl ester of diabetic mice on motor and sensory nerve conduction velocity. Motor and sensory nerve conduction velocities were determined as described in the Materials and Methods section. Data are presented as the mean ± S.E.M. in m/s. The number of mice in each group was the same as shown in Table 2. ^*^ p<0.05 compared to control mice; ^**^ p<0.01 compared to control mice; ^+^ p<0.05 compared to non-treated diabetic mice; ^++^ p<0.01 compared to non-treated diabetic mice.

Effect on nerve conduction velocity, intraepidermal nerve fiber density, thermal nociception and corneal nerve fiber length. Motor and sensory nerve conduction velocities is a common endpoint for examining peripheral neuropathies and was found to be significantly decreased in non-treated diabetic mice (Figure 1).

Treating diabetic mice with menhaden oil significantly improved motor and sensory nerve conduction velocities compared to non-treated diabetic mice. Treating diabetic mice with daily injections of resolvin D1 or E1 significantly improved both motor and sensory nerve conduction velocity with resolvin D1 being more efficacious than resolvin E1. However, motor nerve conduction velocity remained significantly impaired in diabetic mice treated with resolvin D1 or E1 compared to control mice.

Treating diabetic mice with the methyl esters of resolvins D1 or D2 significantly improved motor nerve conduction velocity compared to non-treated diabetic mice but both motor and sensory nerve conduction velocities in these treated mice remained significantly impaired compared to control mice.

The cornea is the most highly innervated part of the human body. The corneal nerves can be visualized using corneal confocal microscopy. This microscope provides a means to perform noninvasive in vivo imaging that allows assessment of the sub-epithelial corneal sensory nerve structure [18,21,22]. It has been proposed that imaging of diabetes-induced changes of these nerves as well as changes in the density of intraepidermal nerve fibers may be surrogate markers for early damage and repair for diabetic peripheral neuropathy [21–25]. We have previously reported that diabetes in rodents causes a decrease in sensory nerve density in the sub-epithelial layer of the cornea as well as a decrease in sensory nerves penetrating the corneal epithelium and the changes caused in the structure and density of corneal nerves by diabetes in rodents is consistent with the changes in these nerves in humans with diabetes [2,17,26–28].

**Figure 2:**
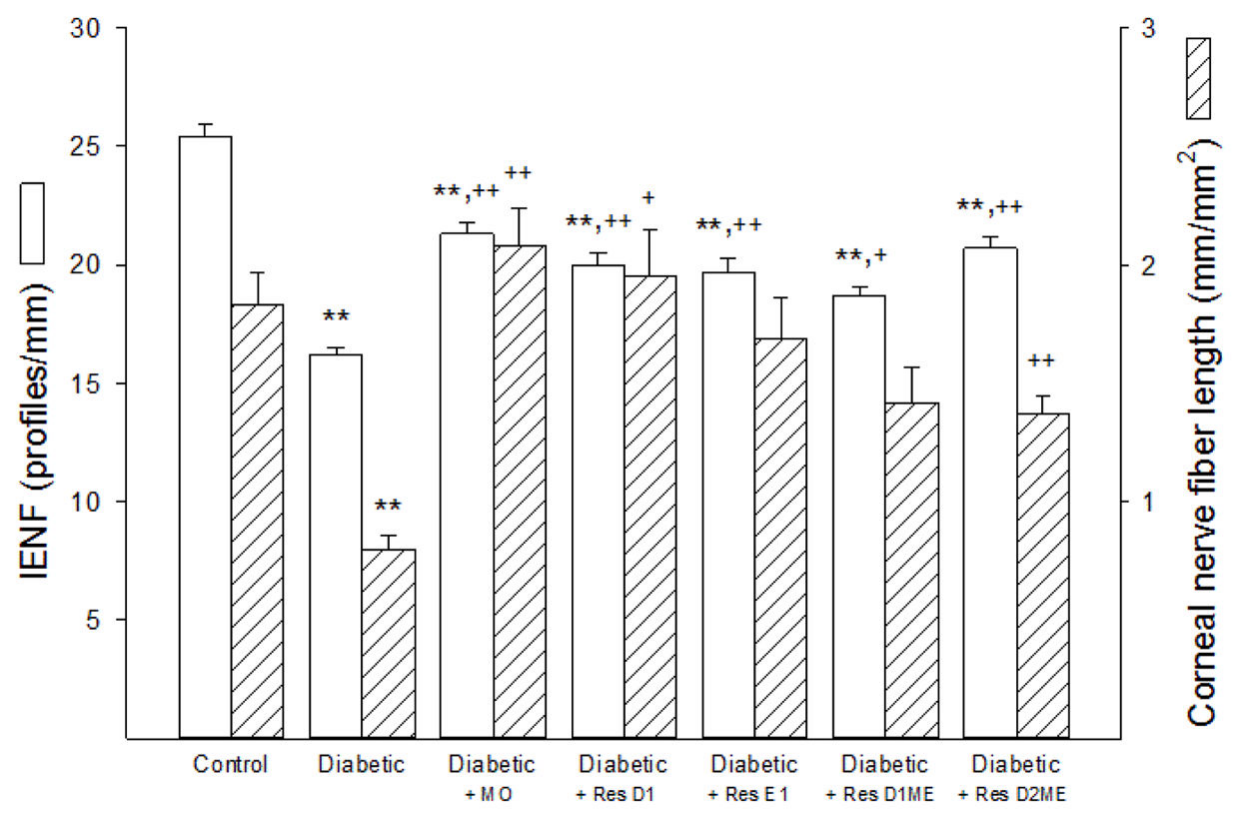
Effect of Menhaden oil dietary enrichment or daily treatment with resolvin D1, E1, D1 methyl ester or D2 methyl ester of diabetic mice on intraepidermal nerve fiber (IENF) density and sub-epithelial cornea nerve fiber length. Intraepidermal nerve fiber density and sub-epithelial cornea nerve fiber length were determined as described in the materials and methods section. Data are presented as the mean ± S.E.M. in profiles/mm and mm/mm^2^, respectively. The number of mice in each group was the same as shown in Table 2. ^**^ p<0.01 compared to control mice; ^+^ p<0.05 compared to non-treated diabetic mice; ^++^ p<0.01 compared to non-treated diabetic mice.

Data in Figure 2 demonstrate that intraepidermal nerve fiber density and sub-epithelial corneal nerve fiber length are significantly decreased in non-treated diabetic mice compared to control mice. Treating diabetic mice with dietary menhaden oil significantly improved intraepidermal nerve fiber density and totally protected corneal nerve fiber density in the sub-epithelial layer compared to non-treated diabetic mice. However, intraepidermal nerve fiber density in diabetic mice treated with dietary menhaden oil remained significantly decreased compared to control mice. Treating diabetic mice with resolvin D1 or E1 also significantly improved intraepidermal nerve fiber density with efficacy that was similar to menhaden oil treatment. Treating diabetic mice with resolvin D1 was more efficacious than resolvin E1 in protecting sub-epithelial corneal nerve fibers. Treating diabetic mice with the methyl esters of resolvins D1 or D2 were about equally effective in protecting intraepidermal nerve fiber density. Methyl esters of resolvin D1 or D2 were also effective in protecting sub-epithelial corneal fiber density but they were generally less efficacious than dietary menhaden oil or exogenous resolvins D1 or E1.

**Figure 3:**
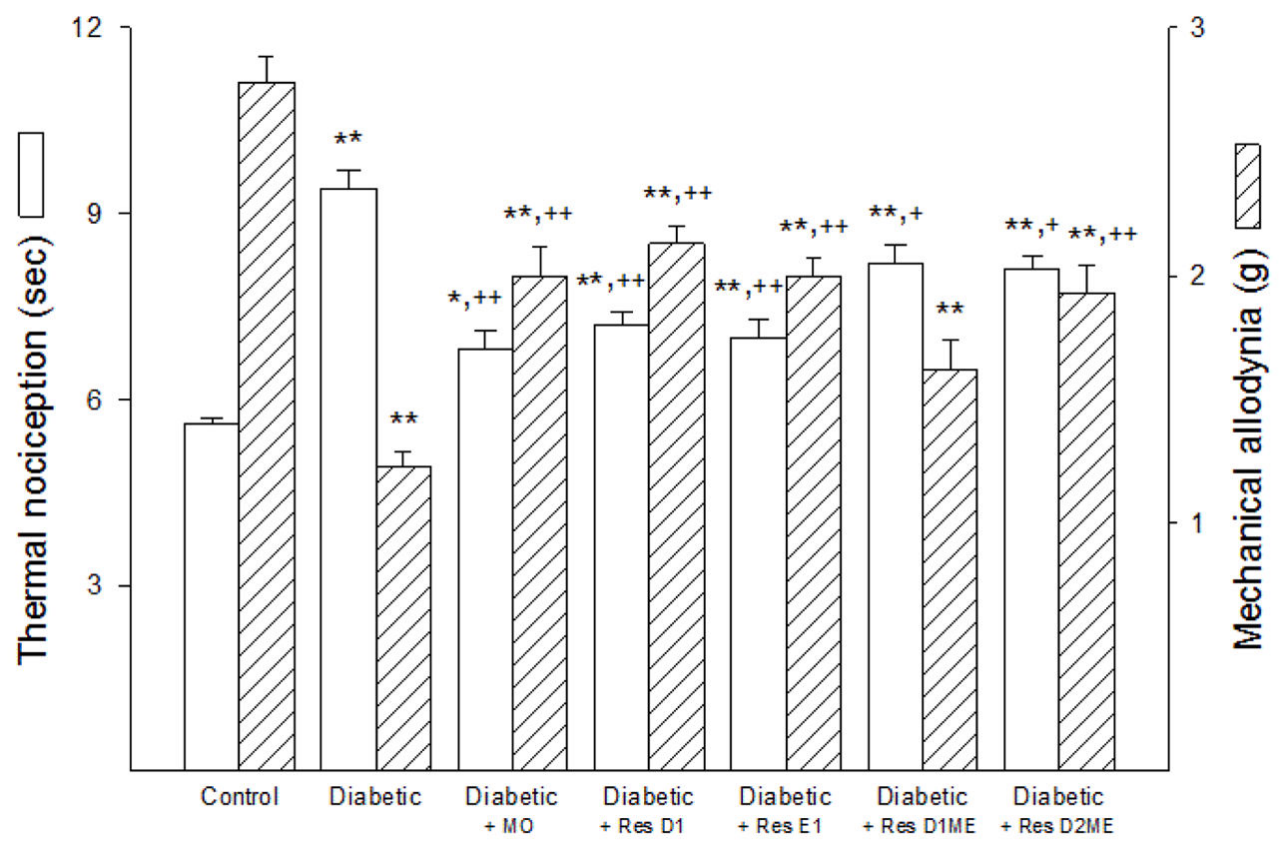
Effect of Menhaden oil dietary enrichment or daily treatment with resolvin D1, E1, D1 methyl ester or D2 methyl ester of diabetic mice on thermal nociception and mechanical allodynia. Thermal nociception and mechanical allodynia were determined as described in materials and methods section. Data are presented as the mean ± S.E.M. in seconds (s) and grams (g), respectively. The number of mice in each group was the same as shown in Table 2. ^*^p<0.05 compared to control mice; ^**^p<0.01 compared to control mice; ^+^ p<0.05 compared to non-treated diabetic mice; ^++^ p<0.01 compared to non-treated diabetic mice.

Data in Figure 3 demonstrate that latency to a thermal stimulus was significantly increased in non-treated diabetic mice compared to control mice. Dietary enrichment of menhaden oil or exogenous treatment with resolvin D1 or E1 reduced the increase in latency to thermal stimulus to a similar degree as compared to non-treated diabetic mice. However, there remained a significant impairment compared to control mice. Treating diabetic mice with the methyl ester of resolvins D1 or D2 was less efficacious toward improving thermal nociception.

Mechanical allodynia was significantly decreased in non-treated diabetic mice compared to control mice, indicating an increase in sensitivity to a mechanical challenge (Figure 3).

Treating diabetic mice with a diet enriched with menhaden oil or with daily injections of resolvin D1, E1 or the methyl ester of resolvin D2 was efficacious in improving mechanical allodynia in diabetic mice. However, mechanical allodynia remained significantly decreased in these treated diabetic mice compared to control mice. We also found that treating diabetic mice with exogenous resolvin D1 methyl ester was the least effective on this endpoint.

## Discussion

The first main finding resulting from these studies was that resolvin E1 was as beneficial as resolvin D1 as an exogenous treatment for diabetic peripheral neuropathy. This indicates that metabolites of either eicosapentaenoic acid (source of E class resolvins) or docosahexaenoic acid (source of D class resolvins) will provide protection from diabetic peripheral neuropathy. The implication is that increased consumption of either or both eicosapentaenoic acid and docosahexaenoic acids, which can be derived from natural sources or through dietary supplementation, i.e., capsules containing fish oil, will be neuroprotective and there is no need to selectively enrich the diet with a sole source of either of these omega-3 polyunsaturated fatty acids. Some studies have found that either eicosapentaenoic acid or docosahexaenoic acid provide a better outcome. It has been reported that eicosapentaenoic acid but not docosahexaenoic acid was associated with significant effects on gene expression involving the interferon pathway as well as down regulation of cAMP responsive element protein 1 and hypoxia inducible factor 1α in human peripheral blood mononuclear cells [29]. The authors concluded that this may relate to the beneficial effects of eicosapentaenoic acid on cardiovascular disease [29]. In contrast, a study performed with dogs showed that docosahexaenoic acid was more effective than eicosapentaenoic acid in attenuating atrial fibrillation vulnerability and atrial remodeling in an experimental model of structural remodeling-induced atrial fibrillation [30]. Both eicosapentaenoic acid and docosahexaenoic acid have been shown to reduce serum triglycerides, but in direct comparison studies docosahexaenoic acid was found to cause a greater reduction than eicosapentaenoic acid and docosahexaenoic acid also raised high-density lipoprotein levels compared to placebo, whereas eicosapentaenoic acid did not [31]. Eicosapentaenoic acid was found to have a greater efficacy compared to docosahexaenoic acid or placebo (coconut oil) as an adjunctive treatment for mild-to-moderate depression [32,33]. However, in our studies exogenous treatment with resolvins D1 or E1 as well as menhaden oil via the diet were efficacious toward multiple endpoints for diabetic neuropathy suggesting a lack of a preferential effect of either eicosapentaenoic acid or docosahexaenoic acid.

The second main finding was that the methyl esters of resolvins D1 or D2 as an exogenous treatment were found not to be more efficacious for diabetic peripheral neuropathy compared to exogenous resolvin D1 even though it has been reported that the half-life of the methyl ester derivatives of resolvins D1 or D2 is longer than the native resolvins [12]. Even if the resolvin methyl esters have a longer half-life in serum they may not be able to reach the target tissues for improving diabetic neuropathy endpoints as effectively as the native compounds. However, this explanation is not true for all tissues since our studies demonstrated that the methyl ester of resolvin D1 and to a lesser extent resolvin D2 methyl ester significantly improved hepatic steatosis.

Anti-inflammatory therapies represent a potential approach for treatment of diabetes complications including neuropathy [34–36]. Sharma and colleagues have shown that inhibiting nuclear factor-κB (NF-κB) using the IκB inhibitor SC-514 or resveratrol provided neuroprotection in diabetic rodents [37–39]. They found that either agent reduced the elevated levels of pro-inflammatory cytokines, inducible nitric oxide synthase and cyclooxygenase-2 (COX-2). Li et al. found that treating diabetic rats with fish oil inhibited mechanical allodynia and thermal hyperalgesia by blocking NF-κB and increasing phosphorylation of protein kinase B (AKT) in dorsal root ganglia [40]. Kellogg et al. has also reported that inhibition of COX-2 provides protection against various diabetic peripheral neuropathy deficits [41]. This study demonstrated that treating type 2 diabetic mice with menhaden oil via the diet or with daily injections of resolvins D1 or E1 provided a similar neuroprotection. Resolvins are potent anti-inflammatory and pro-resolving mediators that are endogenously generated from omega-3 polyunsaturated fatty acids found in fish oils and other marine products and may be a good candidate for the treatment of diabetic neuropathy [42]. Studies in both animals and humans have demonstrated that circulating levels of the resolvins can be increased by dietary supplementation with omega-3 polyunsaturated fatty acids derived from fish oil and resolvin levels can be further enhanced by aspirin [43–46]. In this study we found serum levels of resolvin D1 were increased by nearly 2-fold in diabetic mice treated with a menhaden oil enriched diet compared to serum collected from control mice or untreated diabetic mice. A deficit in the production of resolvins has been demonstrated in obese adipose tissue and restoration of their levels by either exogenous administration or by feeding omega-3 polyunsaturated enriched diets has been shown to improve inflammatory status, insulin sensitivity and ameliorate metabolic dysfunction [42,47–50]. Resolvins have also been shown to be a potential treatment for other inflammatory conditions such as rheumatoid arthritis, inflammatory bowel disease and allergic responses [51–55]. Thus, treatment of diabetic mice with menhaden oil lead to an increase in resolvin production in vivo which would be expected to reduce inflammatory stress and provide a mechanism for improving or protecting from peripheral neuropathy.

In a recent study of subjects with type 1 diabetes it was found that nonalcoholic fatty liver disease, diagnosed by ultrasonography, was strongly associated with an increased risk of distal symmetric polyneuropathy [56]. Therefore, it is reasonable to postulate that a successful treatment for diabetic peripheral neuropathy should also improve nonalcoholic fatty liver disease. Enriching the diet of rodents with menhaden oil increases the eicosapentaenoic acid and docosahexaenoic acid content of the serum and increases the unsaturation index from about 1.5 to 1.9 [2]. In this study enriching the diet of type 2 diabetic mice significantly increased the eicosapentaenoic acid and docosahexaenoic acid content of the liver compared to untreated diabetic mice and decreased the omega-6 to omega-3 fatty acid ratio from 3.1 ± 0.4 in untreated diabetic mice to 0.9 ± 0.2 in treated diabetic mice. Decreasing the omega-6 to omega-3 fatty acid ratio is an indicator of reduced inflammatory stress and has been shown to significantly reduce steatohepatitis [57]. Hepatic steatosis was significantly increased in untreated diabetic mice compared to control mice and was significantly improved with dietary enrichment with menhaden oil or by exogenous treatment with resolvins D1, E1 and the methyl esters of resolvins D1 or D2. Exogenous treatment of diabetic mice with resolvin D1 was the least efficacious but there were no significant differences between the outcomes from any of the treatments. Serum triglyceride and free fatty acid levels were fully corrected with dietary enrichment with menhaden oil or by exogenous treatment with resolvins D1, E1 and the methyl esters of resolvins D1 or D2. In contrast, serum cholesterol levels were not improved with dietary enrichment with menhaden oil or resolvin D2 methyl ester treatment and only partially improved by exogenous treatment of the diabetic mice with resolvins D1, E1 and the methyl ester of resolvin D1. Hepatic insulin resistance is considered to be a central player in the development of metabolic syndrome and nonalcoholic fatty liver disease [58]. Current therapies focus on lifestyle changes and weight loss, but the outcome has been disappointing [58]. Results from our studies suggest that enriching the diet with a source of omega-3 polyunsaturated fatty acids or supplementation with fish oil capsules may improve nonalcoholic fatty liver disease. This is supported by a recent meta-analysis of randomized controlled trials that concluded that supplementation with ω-3 polyunsaturated fatty acids is a practical and effective treatment for nonalcoholic fatty liver disease [59].

## Conclusion

In summary, these studies further demonstrate that dietary supplement with fish oil may provide a safe and efficacious approach to treat diabetic peripheral neuropathy as well as nonalcoholic fatty liver disease and the mechanism responsible for the beneficial effects of fish oil may be associated with the production of resolvins E1 and D1 from eicosapentaenoic and docosahexaenoic acids, respectively.

## Figures and Tables

**Table 1:** Fatty acid % composition of diets measured by gas-liquid chromatography (Data are presented as the mean ± S.E.M. 16:0, palmitic acid; 16:1, palmitoleic acid; 18:0, stearic acid; 18:1, oleic acid; 18:2, linoleic acid; 20:5, eicosapentaenoic acid; 22:6, docosahexaenoic acid. Parentheses indicate the number of experimental determinations).

**Diet**	16:00	16:01	18:00	18:01	18:02	20:05	22:06
**Control 7001 (3)**	15 ± 2	3 ± 1	6 ± 1	22 ± 3	48 ± 3	1 ± 1	<1
**High fat D12492 (3)**	18 ± 2	2 ± 1	9 ± 2	39 ± 4	31 ± 5	<1	<1
**Menhaden oil enriched high fat D10122003 (3)**	18 ± 2	7 ± 1	6 ± 1	23 ± 4	16 ± 2	9 ± 1	9 ± 1

**Table 2:** Effect of Menhaden oil dietary enrichment or daily treatment with resolvin D1, E1, D1 methyl ester or D2 methyl ester of diabetic mice on change in body weight, blood glucose and serum triglycerides, free fatty acids, cholesterol, resolvin D1 and thiobarbituric acid levels and liver proteasome activity and steatosis (Data are presented as the mean ± S.E.M.

Determination	Control (I)	Diabetic (II)	Diabetic+Menhaden Oil (III)	Diabetic+Res D1 (IV)	Diabetic+Res E (V)	Diabetic+Res D1 ME (VI)	Diabetic +Res D2 ME (VII)
	−12	−12	−12	−12	−11	−11	−11
Start weight (g)	25.7 ± 0.4	26.6 ± 0.4	26.5 ± 0.5	26.9 ± 0.4	25.9 ± 0.4	27.1 ± 0.5	25.1 ± 0.5
Weight at Treatment (g)	31.2 ± 0.4	40.7 ± 1.8^a^	38.4 ± 2.1	41.8 ± 2.3^a^	37.2 ± 2.1	40.2 ± 2.0^a^	37.7 ± 1.9
Final weight (g)	33.5 ± 0.6	48.3 ± 3.0^a^	42.5 ± 2.8	46.3 ± 2.9^a^	40.1 ± 3.3	45.6 ± 3.4	40.2 ± 3.4
Blood glucose at Treatment (mg/dL)	139 ± 11	328 ± 27^a^	352 ± 33^a^	351 ± 28^a^	338 ± 37^a^	332 ± 25^a^	397 ± 23^a^
Final Blood glucose (mg/dL)	211 ± 7	416 ± 34^a^	405 ± 38^a^	375 ± 45^a^	380 ± 41^a^	357 ± 44^a^	443 ± 46^a^
Triglycerides (mg/dL)	62 ± 9	100 ± 15	56 ± 8^b^	61 ± 8	58 ± 14	50 ± 6b	56 ± 4^b^
Free fatty acids (mmol/L)	0.44 ± 0.03	0.76 ± 0.14^a^	0.30 ± 0.03^b^	0.35 ± 0.02^b^	0.37 ± 0.06^b^	0.44 ± 0.07^b^	0.42 ± 0.05^b^
Cholesterol (mg/mL)	1.60 ± 0.09	2.91 ± 0.21^a^	3.08 ± 0.12^a^	2.22 ± 0.23	2.09 ± 0.24	2.34 ± 0.21	2.64 ± 0.23^a^
Resolvin D1 (pg/mL)	363 ± 46	396 ± 25	583 ± 72^a^,^b^	ND	ND	ND	ND
Thiobarbituric acid (mg/mL)	0.74 ± 0.05	1.07 ± 0.08^a^	1.18 ± 0.09^a^	0.83 ± 0.07	0.88 ± 0.07	0.94 ± 0.10	0.99 ± 0.03
Liver proteasome activity (U/mg protein)	0.288 ± 0.003	0.199 ± 0.008^a^	0.244 ± 0.006^a^,^b^	0.226 ± 0.009^a^	0.215 ± 0.013^a^	0.215 ± 0.009^a^	0.243 ± 0.010^a^,^b^
Steatosis (% area)	6.7 ± 0.7	57.0 ± 2.2^a^	32.2 ± 1.4^a^,^b^	40.3 ± 1.8^a^,^b^	30.2 ± 2.6^a^,^b^	28.2 ± 2.0^a^,^b^	36.5 ± 1.4^a^,^b^

a P<0.05 compared to control mice;

b P<0.05 compared to diabetic mice.

ND: Not Determined. Parentheses indicate number of experimental).

**Table 3:** Fatty acid % composition of liver measured by gas chromatography (Data are presented as the mean ± S.E.M. 16:0, palmitic acid; 16:1, palmitoleic acid; 18:0, stearic acid; 18:1, oleic acid; 18:2, linoleic acid an omega-6 polyunsaturated fatty acid; 20:4, arachidonic acid an omega-6 polyunsaturated fatty acid; 20:5, eicosapentaenoic acid an omega-3 polyunsaturated fatty acid; 22:6, docosahexaenoic acid an omega-3 polyunsaturated fatty acid. Unsaturation index for Control mice: 1.68 ± 0.04; Diabetic mice: 1.69 ± 0.06; Diabetic+Menhaden oil mice: 1.92 ± 0.08^a^,^b^.

**Condition**	16:00	16:01	18:00	18:01	18:02	20:04	20:05	22:06
**Control (6)**	24 ± 1	3 ± 1	16 ± 2	17 ± 1	13 ± 1	17 ± 1	<1	8 ± 1
**Diabetic (6)**	20 ± 1	2 ± 1	15 ± 1	15 ± 3	17 ± 2	14 ± 1	<1	9 ± 1
**Diabetic+Menhaden oil (6)**	21 ± 1	3 ± 1	14 ± 2	12 ± 2	13 ± 1	6 ± 1^a^,^b^	6 ± 1^a^,^b^	15 ± 1^a^,^b^

Parentheses indicate the number of experimental determinations.

a P<0.05, compared to control mice;

b P<0.05, compared to diabetic mice).
